# Ciguatera Fish Poisoning — New York City, 2010–2011

**Published:** 2013-02-01

**Authors:** Nathan Graber, Faina Stavinsky, Robert Hoffman, Jessica Button, Nancy Clark, Scott Martin, Alison Robertson, John Hustedt

**Affiliations:** New York City Dept of Health and Mental Hygiene; Stony Brook Univ Medical School, Stony Brook, New York; Food and Drug Administration; Public Health Prevention Svc, CDC

During August 2010–July 2011, the New York City Department of Health and Mental Hygiene (DOHMH) received reports of six outbreaks and one single case of ciguatera fish poisoning (CFP), involving a total of 28 persons. CFP results from consumption of certain large, predatory, tropical reef fish that have bioaccumulated ciguatoxins (CTX). CFP is characterized by various gastrointestinal, cardiovascular, and neurologic symptoms. A prolonged period of acute illness can result, and the neurologic symptoms can last months, with variable asymptomatic and symptomatic periods. The first two outbreaks and the single case, involving 13 persons, were reported during August 6–September 13, 2010. DOHMH distributed a health alert in November 2010 requesting health-care providers be alert for CFP signs and symptoms. The health alert resulted in identification of 11 more cases that month and an additional two outbreaks involving four persons in July 2011. In comparison, only four CFP outbreaks, involving 21 persons total, had been reported in New York City (NYC) during the preceding 10 years (2000–2009). DOHMH’s investigation revealed that 13 persons became ill after eating barracuda, and 15 became ill after eating grouper. Although specific and highly sensitive laboratory analyses can detect and confirm CTX in fish, no practical field tests are available for fish monitoring programs. CFP prevention depends on educating the public, seafood suppliers, and distributors about known CFP endemic areas and high-risk fish species. Traceback investigations of fish associated with outbreaks provide valuable information regarding fishing areas associated with CFP. Not all fish from CFP endemic areas are ciguatoxic, but persons who eat fish from endemic regions are at higher risk for CFP. If an illness is suspected to be CFP, public health authorities should be notified and informed of the case history for possible investigation and intervention measures.

On August 6, 2010, an adolescent female aged 16 years, and her mother aged 47 years went to a hospital emergency department (ED) with diarrhea, light-headedness, and perioral tingling after eating barracuda purchased at a fish market in Queens, New York. Hours later, an additional four family members (three males and one female) who had eaten the same fish, reported tingling in their extremities. Two of the four also visited the ED. Later, the four who had gone to the ED experienced abdominal cramps, dizziness, headache, faintness, nausea, and vomiting. Hypotension and bradycardia persisted, despite volume resuscitation with normal saline. The treating physician suspected a link between the barracuda consumption and neurologic and gastrointestinal symptoms (Table 1), subsequently diagnosed CFP,* and contacted the NYC Poison Control Center (PCC). The PCC reported the incident to DOHMH, and a DOHMH inspector collected samples of barracuda from the fish market and the patients’ home. The inspector also embargoed barracuda sale at the fish market.

Samples were analyzed for CTX at the Gulf Coast Seafood Laboratory of the Food and Drug Administration (FDA) using methods developed by FDA to confirm CFP cases. These methods included an in vitro mouse neuroblastoma cell assay for sodium channel toxins to provide a semiquantitative measure of composite ciguatoxicity in fish (1). Extracts that were positive by this method were subsequently analyzed by liquid chromatography–tandem mass spectrometry for unequivocal confirmation of ciguatoxins (1). One meal remnant was confirmed to contain Caribbean CTX-1 and -2 at a toxicity level of 1.1 *μ*g/kg total C-CTX-1 equivalents, more than 10 times the FDA guidance level of 0.1 *μ*g/kg total C-CTX-1 equivalents. The patients reported that some of their neurologic symptoms persisted for 2–5 months (Table 1).

During August–September 2010, an additional seven CFP cases were reported to DOHMH. These consisted of two outbreaks (outbreaks 2 and 3; Table 1) and a single case. All patients experienced symptoms consistent with CFP after eating barracuda purchased from fish markets in three different NYC boroughs and one restaurant (Table 2). On the evening of November 19, 2010, after reading the health alert about CFP, a physician reported a suspected CFP outbreak in Queens (outbreak 4). This new outbreak involved 11 persons from three families who had eaten fish labeled as grouper that was purchased from a Queens supermarket. Five hours after eating the fish, one family member visited the ED with vomiting, nausea, hypotension, and leg cramping. Shortly thereafter, other members of the family reported experiencing numbness and tingling, and two had bradycardia diagnosed several days after fish consumption. In contrast with previously reported cases, four patients experienced tooth pain or paradoxical dysesthesias (Table 1). New York State Department of Agriculture and Markets completed their traceback investigation and identified the same distributor involved in the barracuda-related CFP outbreak reported earlier that year.

On July 12, 2011, two separate outbreaks and an additional four cases that were associated with eating grouper at Manhattan restaurants were reported to DOHMH. One of the patients was a physically active man who swam >2 miles per day before his illness. After the onset of acute CFP symptoms, he had difficulty walking that persisted for several months. A sample of leftover fish was confirmed by FDA to contain 1.9 *μ*g/kg total C-CTX-1 equivalents, exceeding the FDA guidance level by almost 20 times. Before this most recent outbreak, the implicated vendor was inspected by FDA and issued a warning letter detailing violations.

## Editorial Note

CTX are naturally occurring toxins that can accumulate in commonly consumed coral reef fish (e.g., barracuda, grouper, snapper, amberjack, and surgeonfish). Precursors of CTX are derived from marine dinoflagellates (microalgae) that live on the surfaces of seaweeds and denuded corals. These microalgae are consumed by herbivorous fish and undergo bioconversion to the more potent CTX as they move through the food chain. CTX can accumulate in reef fish that eat other fish, reaching levels that can cause CFP among humans when consumed. The toxins are colorless, odorless, tasteless, and temperature-stable, making them difficult to detect or destroy. Consequently, CFP occurrence is not attributable to incorrect food handling, storage, preparation, or procurement methods. The attack rate can be 80%–90% among persons who have eaten a toxic fish, depending on the concentration of CTX in the fish, the total amount of fish consumed, and the consumer’s body weight and health status (2). As in the outbreaks described in this report, symptomatology is variable.

Initial treatment options for CFP are limited and supportive only. The majority of patients experience symptoms within 6–48 hours after eating contaminated fish. In an acutely symptomatic patient, any vital sign instability or electrolyte imbalance should be treated in accordance with the normal standard of care (3). Administration of intravenous mannitol was thought to reduce neuronal edema; however, a randomized double-blind, clinical trial found no evidence of mannitol being superior to normal saline, and mannitol can cause additional side effects, including hypotension, requiring caution during administration (4–6). Treatment of CFP symptoms (e.g., neuropathy, fatigue, and headache) with amitriptyline, sodium channel blockers, and pain medications all have been tried with variable success (4). Consultation with the local PCC is recommended and in NYC fulfills the reporting requirement.

What is already known on this topic?

Ciguatera fish poisoning can occur after eating coral reef fish (e.g., barracuda, grouper, snapper, amberjack, and surgeonfish). Cases are underreported to health authorities, and physicians can have difficulty correctly diagnosing cases, even in areas where poisoning commonly is reported.

What is added by this report?

During August 2010–July 2011, New York City experienced 28 ciguatera fish poisoning cases occurring in six outbreaks and a single case, more than occurred in the previous 10 years combined. Early detection and outreach led to additional cases being identified and treated.

What are the implications for public health practice?

Until the time when premarket testing of fish becomes practical, additional outreach and education to industry and health-care providers is warranted. New York City’s experience from these outbreaks highlights the importance of industry adherence to approved hazard analysis and critical control points plans to reduce the risk for ciguatoxic fish entering the market. This study also illustrates the importance of accurate diagnosis and consistent reporting to public health agencies to ensure the prevention of additional cases through traceback investigations, product embargoes, and regulatory enforcement.

This report reflects the importance of surveillance and outreach networks in responding to patients’ histories, including food consumption, that are indicative of CFP, and highlights prevention challenges. Reports made to the NYC PCC allowed expeditious and effective action when the first cases of CFP were reported. Investigators notified other jurisdictions, consulted local health departments with expertise in CFP prevention and case management, and conducted outreach to NYC health-care providers. In southern Florida, where CFP is endemic, 68% of physicians who were presented with a typical case of CFP diagnosed it correctly (7). As a result of considerable education and outreach efforts by the Florida Department of Health during the past decade, accuracy of CFP diagnosis in that state has improved. However, in other nonendemic regions, diagnostic recognition remains low.

An interstate comparison of reports to PCCs revealed additional trends, beyond the increased number of NYC CFP cases. Unpublished data from CFP-related calls to the American Association of Poison Control Centers during 2000–2010 were analyzed for trends and changes in geographic distribution. The data revealed that the rate of CFP-related calls per capita during 2010, compared with the previous 10 years, was 55% higher in NYC but 44% lower in Florida. Although this data set might not be representative of individual state CFP records, the rate per capita of U.S. cases remained relatively constant throughout the preceding 11 years. This increase of reported cases in NYC might reflect changing sources and diversity of fish species marketed in NYC and elsewhere. The increase might also indicate improved awareness and capacity for investigation by the medical and public health community. The decrease in CFP reports from Florida likely was the result of improved awareness of CFP after extensive long-term outreach and education efforts and specific guidance on the harvest of high-risk fish in this endemic region.

CFP is considered a highly underreported illness, with only an estimated 10% of cases reported to health authorities (7). Increasing awareness among health-care providers might improve reporting and investigation. However, CFP prevention is complicated by difficulty in identifying high-risk fishing grounds and inadequate industry knowledge and compliance with the FDA seafood Hazard Analysis and Critical Control Point (HACCP) regulations.† Premarket testing of fish for CTX is not feasible because of the lack of rapid field methods and the sporadic distribution of toxic fish, even in endemic areas. Coordinated tracebacks of implicated fish by federal and state agencies to specific fishing grounds remains the primary strategy for managing CFP.

The findings in this report are subject to at least three limitations. First, meal remnant samples were available only in three of the six CFP outbreaks. Second, where physician reports to the PCC were unavailable, the symptoms were based entirely on self-report or secondhand reports from family members. Finally, additional cases might have occurred but were unrecognized because of lack of physician awareness to make an appropriate diagnosis and the need to report.

This investigation demonstrates the value of CFP-implicated fish traceback along with updated information on emerging CFP risks, including new harvest areas and species. Prevention through education alone might be limited by seafood mislabeling. Reports indicate that 20%–25% of all seafood products are mislabeled (8). A recent assessment of seafood purchased at retail stores and restaurants in New York, New Jersey, and Connecticut indicated that >20% of 190 specimens were mislabeled, incompletely labeled, or misidentified by employees (8). Methods for fish species identification using DNA barcoding have been validated (9) and are being implemented in several U.S. state and federal laboratories, as well as academic institutions. These methods have been applied to multiple CFP cases. Ongoing collaborative efforts with federal, state, and local agencies tasked with consumer protection and food safety might be useful in controlling CFP and mislabeling of fish (10). Until accurate and cost-effective means of premarket testing become available, prevention of additional cases will continue to be dependent on HACCP compliance by the seafood industry and CFP diagnosis and reporting by health-care providers, warranting additional outreach and education.

## Figures and Tables

**TABLE 1:** Characteristics of persons with suspected ciguatera fish poisoning — New York City, August 2010–July 2011

Patient	Outbreak	Date fish consumed	Age (yrs)	Sex	Hours from consumption to symptom onset	Reported fish consumed*	Sought medical attention	Hospitalized	Symptoms			

									Gastrointestinal	Cardiac	Neurologic	Nonspecific/Other
1	1	Aug 6, 2010	47	F	7	Barracuda	Yes	Yes (ICU)	N, V, D, CR	S, B, LBP,	NT, P	ST, DZ
2	1	Aug 6, 2010	16	F	7	Barracuda	Yes	Yes (ICU)	N, V, D, CR	S, B, LBP, HP	DW	CS, W, DZ
3	1	Aug 6, 2010	50	M	8	Barracuda	Yes	No	N, V, D, CR	S, LBP, HP	NT, DW	CS, W, DZ
4	1	Aug 6, 2010	31	M	8	Barracuda	Yes	No	N, V, D, CR	S, LBP, HP	NT, DW	CS, W, DZ
5	1	Aug 6, 2010	12	F	8	Barracuda	Yes	No	CR		NT	H
6	1	Aug 6, 2010	24	M	3	Barracuda	Yes	No	D, CR		NT, P	R
7	2	Aug 16, 2010	43	F	3.5	Barracuda	Yes	No	N, V, D, CR		DW, P	CH, My
8	2	Aug 16, 2010	49	F	4.5	Barracuda	Yes	No	N, V, D, CR	B	P	CH
9	None	Sep 14, 2010	50	M	2	Barracuda	Yes	No	D	B		My, W
10	3	Aug 24, 2010	32	F	20	Barracuda	Yes	Yes	N, V, D		DW, P	My, DZ, Fv
11	3	Aug 24, 2010	31	M	11	Barracuda	No	No	D		DW, P	My, W, DZ, Fv
12	3	Aug 24, 2010	33	F	N/A	Barracuda	No	No			P	My
13	3	Aug 24, 2010	41	M	N/A	Barracuda	No	No				My
14	4	Nov 13, 2010	2	F	0.5	Grouper	Yes	No	N, V, CR		NT	Fv
15	4	Nov 9, 2010	28	F	38.5	Grouper	No	No	D		NT	W
16	4	Nov 9, 2010	33	F	15	Grouper	No	No	N, V		NT	W
17	4	Nov 9, 2010	56	F	3.5	Grouper	Yes	No	N, V, D, CR	LBP, HP, B	NT, PD, DW, P	W, H
18	4	Nov 9, 2010	32	F	25.5	Grouper	Yes	No	N, V, D, CR		NT, TP, PD, DW, P	W, Fv, H
19	4	Nov 9, 2010	58	M	4.5	Grouper	No	No	D, CR	HP	NT, PD, DW, P	W, H
20	4	Nov 9, 2010	12	F	1	Grouper	Yes	No	CR		NT, DW, P	W
21	4	Nov 9, 2010	53	F	44.5	Grouper	Yes	No	D		NT, DW, P	W, H
22	4	Nov 9, 2010	7	F	4.5	Grouper	Yes	No	N, V, D, CR		NT, P	W, H
23	4	Nov 9, 2010	7	F	16.5	Grouper	Yes	No	N, V, CR		NT, P	H
24	4	Nov 19, 2010	51	M	10	Grouper	Yes	No	N, V, D, CR	HP, LBP, B	NT, TP, DW, P	W
25	5	Jul 13, 2011	54	F	9	Grouper	No	No	N, CR			
26	5	Jul 13, 2011	51	M	5	Grouper	No	No	N, D, CR		PD, P	W
27	6	Jul 13, 2011	48	F	4	Grouper	Yes	Yes	N, V, D, CR		PD	
28	6	Jul 13, 2011	60	M	6	Grouper	No	No	D, CR		PD	W

**Abbreviations:** B = bradycardia; CH = chills; CR = cramps; CS = cold sweats; D = diarrhea; DW = difficulty walking; DZ = dizziness; F = female; Fv = fever; H = headache; HP = heart palpitations; ICU = intensive-care unit; LBP = hypotension; M = male; My = myalgia; N = nausea; N/A = not available; NT = numbness or tingling; P = pruritus; PD = paradoxical dysesthesias; R = rash; S = syncope; ST = swollen tongue; TP = tooth pain; V = vomiting; W = weakness.

* None of the fish were speciated; all species were reported from food establishment records.

**TABLE 2:** Frequency of reported symptoms among ciguatera patients (N = 28) — New York City, August 2010–July 2011

Symptom	No.	(%)
Cramps	20	(71)
Diarrhea	20	(71)
Nausea	17	(61)
Weakness	16	(57)
Pruritus	16	(57)
Numbness/Tingling	16	(57)
Vomiting	15	(54)
Difficulty walking	12	(43)
Headache	7	(25)
Myalgia	6	(21)
Dizziness	6	(21)
Paradoxical dysesthesias	6	(21)
Heart palpitations	6	(21)
Bradycardia	6	(21)
Hypotension	6	(21)

## Footnotes

* Additional information on CFP signs and symptoms available at http://www.nyc.gov/html/doh/downloads/pdf/cd/2010/10md25.pdf.

† Additional information, including advisories and guidance related to high-risk species and endemic regions, is available at http://www.fda.gov/food/foodsafety/hazardanalysiscriticalcontrolpointshaccp/seafoodhaccp/default.htm.
